# Bayesian Networks Analysis of Malocclusion Data

**DOI:** 10.1038/s41598-017-15293-w

**Published:** 2017-11-10

**Authors:** Marco Scutari, Pietro Auconi, Guido Caldarelli, Lorenzo Franchi

**Affiliations:** 1 Department of Statistics, University of Oxford, 24-29 St Giles’, Oxford, OX1 3LB UK; 2 Private Practice of Orthodontics, Roma, Italy; 3 IMT School for Advanced Studies, Piazza San Francesco 19, 55100 Lucca, Italy; 4 Istituto dei Sistemi Complessi CNR, Unità Sapienza, Dip. Fisica, P.le A. Moro 2, 00185 Rome, Italy; 5 London Institute for Mathematical Sciences, 35a South St, Mayfair, London, W1K 2XF UK; 6 Dipartimento di Chirurgia e Medicina Traslazionale, Università degli Studi di Firenze, Firenze, Italy; 7 Thomas M. Graber Visiting Scholar, Department of Orthodontics and Pediatric Dentistry, School of Dentistry, The University of Michigan, Ann Arbor, MI USA

## Abstract

In this paper we use Bayesian networks to determine and visualise the interactions among various Class III malocclusion maxillofacial features during growth and treatment. We start from a sample of 143 patients characterised through a series of a maximum of 21 different craniofacial features. We estimate a network model from these data and we test its consistency by verifying some commonly accepted hypotheses on the evolution of these disharmonies by means of Bayesian statistics. We show that untreated subjects develop different Class III craniofacial growth patterns as compared to patients submitted to orthodontic treatment with rapid maxillary expansion and facemask therapy. Among treated patients the *CoA* segment (the maxillary length) and the *ANB* angle (the antero-posterior relation of the maxilla to the mandible) seem to be the skeletal subspaces that receive the main effect of the treatment.

## Introduction

The use of statistical methods in medicine is crucial to overcome the large individual variability in the pathological features of different patients^1^. In the orthodontic discipline, the variability of craniofacial disharmonies is especially relevant due to important differences between individuals in the amount and direction of facial growth due to heredity, gender, ethnic background, and functional characteristics^2^. In this paper we introduce an approach based on modern techniques from Bayesian statistics for Complex Network analysis to estimate and describe the evolution of orthodontic features measured simultaneously on a set of patients. An incredibly large amount of integrations of the various components of the craniomaxillary and mandibular combinations are possible during the growth process: the integration of these features determines the ultimate dentofacial harmony or disharmony^3^. An in-depth understanding of the resulting large amounts of interrelated data obtained from clinical, radiographic, and functional analyses is required to establish a solid knowledge basis for orthodontic diagnoses. Malocclusions are isoforms of disharmony: they express a form of organic integrity during the growth process by assimilating existing elements in a new synthesis. These isoforms incur costs in terms of weakness of mechanotrasduction, cumulative occlusal trauma, adaptability, local optimisation, competition between tooth elements for space, and outcome uncertainty about the ultimate facial appearance^4^. These conditions are rarely a consequence of an abnormality in a single craniofacial component, so individual clinical and radiological measurements are likely to be less indicative than the interplay between the measurements themselves. In the case of patients affected by Class III malocclusion (characterised by the protrusion of lower dental arch), skeletal imbalance is established early in life, becomes more pronounced during puberty, and continues to increase until skeletal maturation is complete^4^. Therefore, predicting treatment success or failure early in a single Class III patient based on a small number of morphometric determinants is problematic^5^.

Here we present a methodology that makes use of longitudinal data collected from a sample of orthodontic patients to evaluate possible causal paths linking orthodontical features during the growth process and the changes in those paths induced by the treatment. Practising orthodontists often perform clinical reasoning under uncertainty about facial growth, with incomplete information, receiving far more inputs than they can consciously consider; and as a result they are forced to distil clinical and/or radiological evidence into regularities and patterns^6^. Modern techniques in computational statistics build on fundamental principles of probability theory^7^ to provide a better understanding and visualisation of complex data by learning those regularities and patterns directly from the data, thus producing rigorous yet tractable models of domains in which expensive computations are required for quantitative reasoning^8^. In particular, Bayesian statistics^9,10^ develops the idea of combining the information contained in experimental data with prior knowledge available from the literature and from previous experiments to evaluate the probability of specific hypotheses; an approach that is natural and especially useful in biological and medical research^11,12^. These computational tools can summarise a biological system involving multiple interacting components into a simplified representation that captures the interplay between those components; and that can provide insights on how those components influence (and possibly are causal for) each other^13^. A convenient device to represent such complex patterns of relationships are complex networks^14^, which provide a high-level, abstracted view of the interplay between the variables of interest by representing them as nodes and by linking them with arcs that show how those variables interact with each other. A popular choice for this kind of representation are Directed Acyclic Graphs (DAGs), in which links represent direct probabilistic dependencies and have arrows indicating the direction of the dependence. In this paper we intend to develop such a model in the context of orthodontics, combining DAGs with the joint probability distribution of the craniofacial variables of interest^15^. The contribution of the DAG in this case is to visualise the set of relationships between these variables and to determine how they may be grouped into communities. Networks have already been used in the literature to describe the evolution of patients with malocclusions^16^ and to help in the formulation of diagnosis^17^. Indeed, the exact focal morphological areas of the treatment effect and treatment priorities are both still under discussion in the clinical orthodontics community.

Unfortunately, current clinical evidence has been unable to fully elucidate the network of causalities that link the relevant skeletal components, the starting point of the treatment, treatment priorities, and the best way to channel and disseminate the effects of the treatment^17^. Here we will try to address such questions for Class III malocclusion, a dysmorphosis characterised by growth excess of the mandible and/or a defective growth of the maxilla, with protrusion of the lower dental arch. To this end we will use a set of 147 longitudinal measurements of various craniofacial features on 143 Class III growing patients evaluated at least twice between the ages of 6 and 19. Sixty-six of these subjects were undergoing orthodontic treatment by early rapid maxillary expansion and facemask therapy followed by fixed appliances, while the remaining 77 were not subject to any treatment. We will estimate Bayesian networks from these data and we will use resampling techniques from modern statistics to produce a consensus network model that describes the relationships between treatment and craniofacial figures and to evaluate its predictive accuracy. We find the resulting network to be consistent with a number of key characteristics of Class III malocclusion as known from current clinical evidence and literature, which we use to validate the relationships we learn from the longitudinal data. Furthermore, the network displays good predictive accuracy for the dynamics of Class III malocclusion in new patients. Finally, we use the network to identify the focal morphological areas of the treatment effect on the basis of the causal relationships captured by the network structure.

## Methods

### The Data

The data contain longitudinal measurements on a set of 147 Class III growing patients (83 female, 60 male) evaluated at least twice between the ages of 6 and 19. Two sets of simultaneous measurements at ages ${T}_{1}$ (6 to 19 years, average $8\pm 1$ years) and ${T}_{2}$ (5 to 19 years, average $15\pm 1$ years) are available for all patients, in addition to a *Treatment* variable identifying treated from untreated patients. For each untreated subject, a *Growth* variable indicating the prognosis as positive or negative in comparison with the normal craniofacial progression was reported. The complete list and details for the 8 variables for this data set can be found in the Supplementary Information.

### Correlation Networks

We represent the entire craniofacial system as an aggregate structure of a variety of agents where the clinical (e.g., radiographic, functional) features are the vertices of a network whose edges are the relationships between them. To build the network we start from a measure of correlation among the cephalometric variables ${X}_{a},{X}_{b}$ and in particular we compute the Pearson correlation coefficient *r* defined as

1 $${r}_{ab}=\frac{{\sum }_{i=1}^{n}({X}_{a}(i)-{\overline{X}}_{a})({X}_{b}(i)-{\overline{X}}_{b})}{\sqrt{{\sum }_{i=1}^{n}{({X}_{a}(i)-{\overline{X}}_{a})}^{2}}\sqrt{{\sum }_{i=1}^{n}{({X}_{b}(i)-{\overline{X}}_{b})}^{2}}}$$

where ${X}_{a}(i)$ is the *i*-th value of the feature ${X}_{a}$ as observed in the data and ${\overline{X}}_{a}$ is the arithmetic mean of the *n* values observed for ${X}_{a}$, *e.g*. ${\overline{X}}_{a}=({\sum }_{i=1}^{n}{X}_{a}(i))/n$. Pairs of variables with values of $|{r}_{ab}|$ above the threshold of 0.4 are linked by edges in the correlation network.

### Bayesian Statistics

The field of statistics provides several approaches to estimate the probability of particular events of interest and to model the laws that govern the phenomena under investigation. For instance, we can estimate the former with its observed, empirical frequency (*frequentist statistics*); and the latter by making assumptions on the distribution of the data and estimating the values of the parameters of the model as those that are best supported by the data (that is, having the *maximum likelihood*)^18^. A second approach is given by *Bayesian statistics* ^19^, in which we also assume *a priori* a distribution for the parameters of the model. That distribution is then updated based on the observed data to reflect the current understanding of the phenomenon; the result is the *posterior distribution* of the parameters given the data. For instance, consider the probability $p(e|{c}_{i})$ of the occurrence of an event *e* we observe under one of several possible conditions ${c}_{i},i=\mathrm{1,}\ldots ,k$. A classic approach in statistics is to estimate $p(e|{c}_{i})$ by means of its frequency (the ratio of how many times the event is observed over the total number of measurements), and then to diagnose the condition as that that has the largest $p(e|{c}_{i})$. On the other hand, Bayesian statistics answers a different question, what is the probability of each condition ${c}_{i}$ given the event $e$? Using Bayes theorem, we can write

2 $$p({c}_{i}|e)=\frac{p(e,{c}_{i})}{p(e)}=\frac{p({c}_{i})p(e|{c}_{i})}{p(e)}=\frac{p({c}_{i})p(e|{c}_{i})}{{\sum }_{j=1}^{k}p({c}_{j})p(e|{c}_{j})}$$

to express the *a posteriori* probability of ${c}_{i}$ as a function of the *a priori* probability $p({c}_{i})$ of the condition and $p(e|{c}_{i})$ relative that of the complete set of conditions. As a result, we obtain a completely specified probabilistic model we can use to test experimental hypotheses and that can easily incorporate additional information available from external sources via the $p({c}_{i})$ terms. Importantly, this makes it possible to iteratively update $p({c}_{i}|e)$ as new data becomes available by taking the current estimates of $p({c}_{i}|e)$ as the *a priori* $p({c}_{i})$ for the new data to compute new, up-to-date estimates of $p({c}_{i}|e)$.

### Differential Equations Models

Since we are interested in modelling the evolution of malocclusion and its response to treatment over time, we will model the data using the differences of the craniofacial features between different time points instead of the raw point measurements. We assume that each difference can be modelled with a linear regression^20^ of the form

3 $${\rm{\Delta }}Y=\mu +{\rm{\Delta }}T{\beta }_{1}+{\rm{\Delta }}{X}_{1}{\beta }_{2}+\ldots +{\varepsilon }_{{\rm{\Delta }}Y}$$

where ${\varepsilon }_{{\rm{\Delta }}Y}\sim N(\mathrm{0,}\,{\sigma }_{{\rm{\Delta }}Y}^{2})$, ${\rm{\Delta }}T={T}_{2}\,-\,{T}_{1}$ and ${\rm{\Delta }}Y={Y}_{{T}_{2}}\,-\,{Y}_{{T}_{1}}$ and so forth for the other regressors. We can then rewrite Eq. 3 as

4 $$\frac{{\rm{\Delta }}Y}{{\rm{\Delta }}T}={\mu }^{\ast }+\frac{{\rm{\Delta }}{X}_{1}}{{\rm{\Delta }}T}{\beta }_{2}^{\ast }+\ldots +{\varepsilon }_{\frac{{\rm{\Delta }}Y}{{\rm{\Delta }}T}}$$

which in the limit of ${\rm{\Delta }}T\to 0$ can be considered as a set of differential equations that models the rates of change. (This is a particular case of structural equation models^21^, which are widely used in statistical genetics and systems biology^22,23^.) The relationships between the differences are assumed to be well approximated by a linear behaviour. This constraint is intrinsically enforced by the data: only 120 out of 147 patients have been measured only twice, making it impossible to estimate any trend more complex than linear. Note that Eqs 3 and 4 imply that craniofacial features change linearly over time, because each rate of change ${\rm{\Delta }}Y/{\rm{\Delta }}T$ depends on the rates of change of other variables but not on time itself. To have a nonlinear trend we would need

5 $${\rm{\Delta }}Y=\mu +{\rm{\Delta }}T{\beta }_{1}+{({\rm{\Delta }}T)}^{2}{\beta }_{2}+\ldots \Rightarrow \frac{{\rm{\Delta }}Y}{{\rm{\Delta }}T}={\mu }^{\ast }+{\rm{\Delta }}T{\beta }_{2}^{\ast }+\ldots \Rightarrow \frac{{\rm{\Delta }}Y}{{\rm{\Delta }}{T}^{2}}={\beta }_{2}^{\ast }\ne 0.$$

Furthermore, including the *Growth* and *Treatment* in the differential equations makes it possible to have regression models of the form

6 $$\frac{{\rm{\Delta }}Y}{{\rm{\Delta }}T}={\mu }^{\ast }+\frac{{\rm{Growth}}}{{\rm{\Delta }}T}{\beta }_{G}^{\ast }+\frac{{\rm{Treatment}}}{{\rm{\Delta }}T}{\beta }_{TR}^{\ast }+\frac{{\rm{\Delta }}{X}_{1}}{{\rm{\Delta }}T}{\beta }_{2}^{\ast }+\ldots +{\varepsilon }_{\frac{{\rm{\Delta }}Y}{{\rm{\Delta }}T}}$$

thus allowing for different rates of change depending on whether the patient shows positive developments or not in the malocclusion and whether he is being treated or not. Conversely, we do not allow the treatment level to depend on ${\rm{\Delta }}T$, since patients are either treated or untreated for the whole period of observation; and we do not assign a regression model to ${\rm{\Delta }}T$ because we assume that it does depend on any measured variables.

### Bayesian Networks

A Bayesian network^24,25^ is a statistical model to describe probabilistic relationships among a set of variables using a directed acyclic graph (DAG). The global distribution of the variables ${\bf{X}}=\{{X}_{1},\ldots ,{X}_{N}\}$, where *N* is the number of different features (in this case $N=8$), is decomposed into a the local distributions of the individual variables ${X}_{i}$ as

7 $$p({\bf{X}})=\prod _{i=1}^{N}p({X}_{i}|Pa({X}_{i}))$$

where $Pa({X}_{i})$ are the variables that correspond to the parents of ${X}_{i}$ in the DAG (i.e. the nodes with an arc pointing towards ${X}_{i}$). The process of estimating such model is called *learning*, and consists in two steps:

- “learning” which arcs are present in the graph (i.e. which probabilistic relationships are supported by the data);
- “learning” the parameters that regulate the strength of those dependencies.
  The former is known as *structure learning*, and the latter as *parameter learning*. In the context of the differential equations described above in Eq. 3, in structure learning we determine which regressors (if any) are present in each differential, while in parameter learning we estimate the values of the corresponding regression coefficients. In order to do that, we assume that the errors in each differential equation (represented by the ${\varepsilon }_{{\rm{\Delta }}Y}$ term) are normally distributed, independent, homoscedastic and with mean zero. Under these assumptions, each differential equation can be treated as a classic linear regression model and estimated by ordinary least squares^26^; and the regressors correspond to the variables associated to the nodes that are parents of ${\rm{\Delta }}Y$ in the DAG.
  Structure learning is similarly based on model selection procedures for classic regression models. Since we operate in a Bayesian setting, we select which variables are statistically significant regressors in each differential equation as those that maximise the posterior probability of the Bayesian network, which we approximate with the *Bayesian Information Criterion* ^27^. Those regressors are the parents of the node corresponding to the response variable in the DAG, and are chosen using hill-climbing, a greedy search algorithm based on step-wise selection^28^. The only restriction imposed by Bayesian networks is that, once the probabilistic relationships are represented as a directed graph, the graph should be acyclic.
  While it is possible in principle to learn all dependencies from the data, Bayesian networks can easily include prior knowledge available from the literature and the practice of the discipline to produce more informative models and to overcome the inherent noisiness of orthodontic data. This can be done by encoding the available prior knowledge in sets of *whitelisted arcs* (which we know represent real dependencies and thus should be forced to be present in the graph) and *blacklisted arcs* (which correspond to relationships we know to be impossible). In particular:
- Craniofacial features do not determine ${\rm{\Delta }}T$ or *Treatment*, so we blacklist any arc from the former to the latter. We also blacklist any arc from the craniofacial features to *Growth*, as we interpret them to be determined by the overall evolution of the malocclusion (including unobserved factors) as expressed by *Growth*. This also leads to a more intuitive parameterisation of the differential equations, with different regimes for the craniofacial features depending on the prognosis.
- We blacklist any arc from ${\rm{\Delta }}T$ and *Treatment* as discussed above.
- We whitelist the dependence structure ${\rm{\Delta }}ANB\to {\rm{\Delta }}IMPA\leftarrow {\rm{\Delta }}PPPM$ ^1,4,5^.
- We whitelist the arc from ${\rm{\Delta }}T$ to *Growth* to allow the prognosis to change over time.

We want to clarify the meaning of the variable *Growth* we use in our analysis: *Growth* reflects the expected prognosis of the patient at the time of the visit. In such respect this is a “static” variable and does not reflect a measure of the effective growth. Quality of growth has been evaluated by considering the normal evolution of the maxillomandibular sagittal imbalance (CoGn-CoA) with respect to average population. Patients near the average values were diagnosed as “good growers” while the others were indicated as “bad growers”^29^. Furthermore, to reduce the impact of the noise present in the data, we use a second Bayesian technique called *model averaging* to improve the reliability of structure learning^30^. Typically, to examine the phenomenon under investigation we estimate a single model from the data, and we draw our conclusions from that model treating it as a “fixed” quantity. In doing so we underestimate the degree of uncertainty present in those statistical conclusions by ignoring the fact that the estimated model is not “fixed”, but carries its own uncertainty from the selection procedure used to learn it from the data^31^. Intuitively, we can imagine that adding or removing a few observations from the data may result in a different model being identified, in turn leading to different conclusions. To reduce this model uncertainty, we re-sample the data 200 times using bootstrap^32^ and we perform structure learning separately on each of the resulting samples, thus collecting 200 DAGs. We then compute the frequency with which each appears in those 200 graphs, known as the *arc strength*, and we compute an “average”, consensus DAG by selecting those arcs that have a frequency above a certain threshold. (The threshold can either be estimated from the data or set to an arbitrary value, such as 0.85 below, for the purpose of obtaining a sparse DAG that is easier to interpret.) The averaged Bayesian network model has a number of favourable statistical properties; in particular, it is less sensitive to noisy data and typically produces more accurate predictions for new observations.

Once we have estimated the average Bayesian network and the values of the regression coefficients in the differential equations it describes, we evaluate its predictive accuracy using 10-fold cross-validation^33^. 10-fold cross-validation is a model validation technique that assesses how well a statistical model generalises to independent data or, in other words, how accurately it will predict the behaviour of new observations. It is implemented as follows.

1. We split the data into 10 subsets (called *folds*) of the same size (or as close as possible).
2. For each fold in turn:
  - we take that fold as the *test set*;
  - we take the rest of the data as the *training set*;
  - we learn the Bayesian network model on the training set, both the structure and the parameters;
  - we predict each variable in turn for the observations in the test set, from the model we learned from the training set and from all the other variables in the test set;
3. We collect the pairs of (observed, predicted) values for all the observations and:
  - for each continuous variable, we compute the correlation between the observed and predicted pairs (this quantity is called *predictive correlation*);
  - for Growth, we compute the number of misclassified predicted values using the observed values as the true values (this is the *predictive classification error*, which the complement of predictive accuracy).

In addition, we use the averaged Bayesian network for inference to answer a number of crucial questions and to check whether it reflects the available knowledge of how the measured variables interact with each other and with the treatment. In the context of Bayesian networks, this is typically done using a technique called *belief updating*, in which we estimate the posterior probability of a certain event or the posterior estimate of some parameter conditional on some evidence on the values of one or more variables. Several exact and approximate approaches are available in the literature^25^; in this paper we use logic sampling for its simplicity. Logic sampling is defined as follows:

1. Define a counter ${n}_{{\bf{E}}}=0$ for the evidence and a counter ${n}_{{\bf{q}}}=0$ for the event of interest;
2. For a suitably large number of times (10^4^–10^6^):
  - generate a random sample from the Bayesian network.
  - if the random sample matches the evidence, set ${n}_{{\bf{E}}}={n}_{{\bf{E}}}+1$;
  - if the random sample matched both the evidence and the event, set ${n}_{{\bf{E}},{\bf{q}}}={n}_{{\bf{E}},{\bf{q}}}+1$.
3. Estimate the conditional probability of the event given the evidence with ${n}_{{\bf{E}},{\bf{q}}}/{n}_{{\bf{E}}}$; or compute the posterior estimates of the parameters of interest using those random observations that match the event.

By applying this approach we can answer arbitrary questions, which are called *conditional probability queries*, from a Bayesian network. We perform the whole analysis using the bnlearn package^15^ for R^34^.

## Results

### Raw data

**Figure 1:**
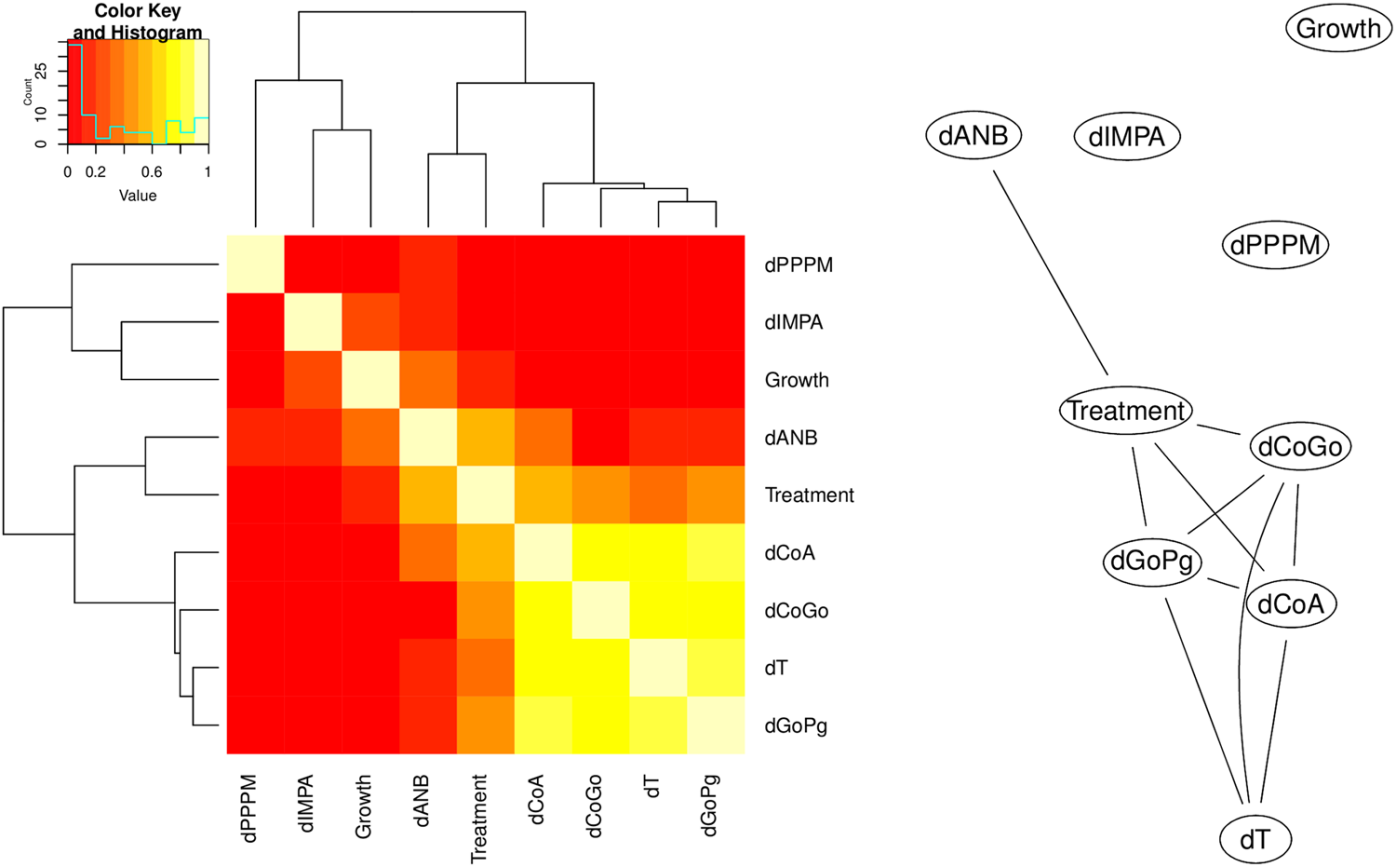
(left) A heatmap of the Pearson’s correlation between variables measured on the patients. (right) The correlation network displaying Pearson’s correlations greater than 0.40 in absolute value.

As discussed in the literature^16,17^, craniofacial features evolve and respond to external stimuli as a system and therefore they form clusters of variables with high (greater than 0.40 in absolute value) Pearson’s correlation. We observe this phenomenon in Fig. 1 (right panel), in which all the craniofacial features are connected with the exception of ${\rm{\Delta }}IMPA$ and ${\rm{\Delta }}PPPM$. *Treatment* is also connected to four of these features, and presents similar correlations values with all of them (Fig. 1, left panel), making it difficult to establish the focal point of its action.

**Figure 2:**
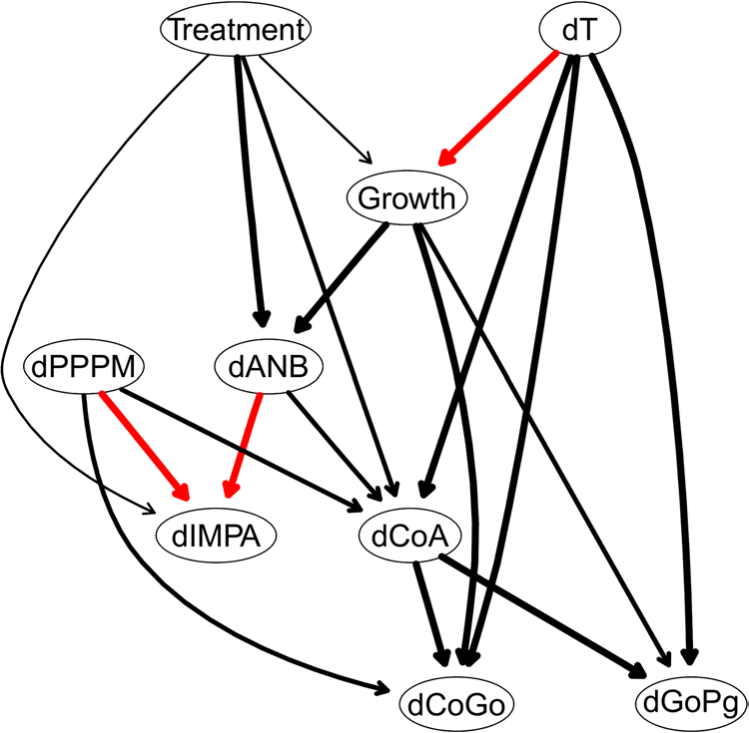
The DAG underlying the consensus Bayesian network learned from the variables measured on all 143 patients. Arcs in red are constrained to be present in the network by the whitelist. The thickness of the arcs is in the proportion to their strength; only arcs with a strength greater than 0.5 are included in the consensus network.

**Figure 3:**
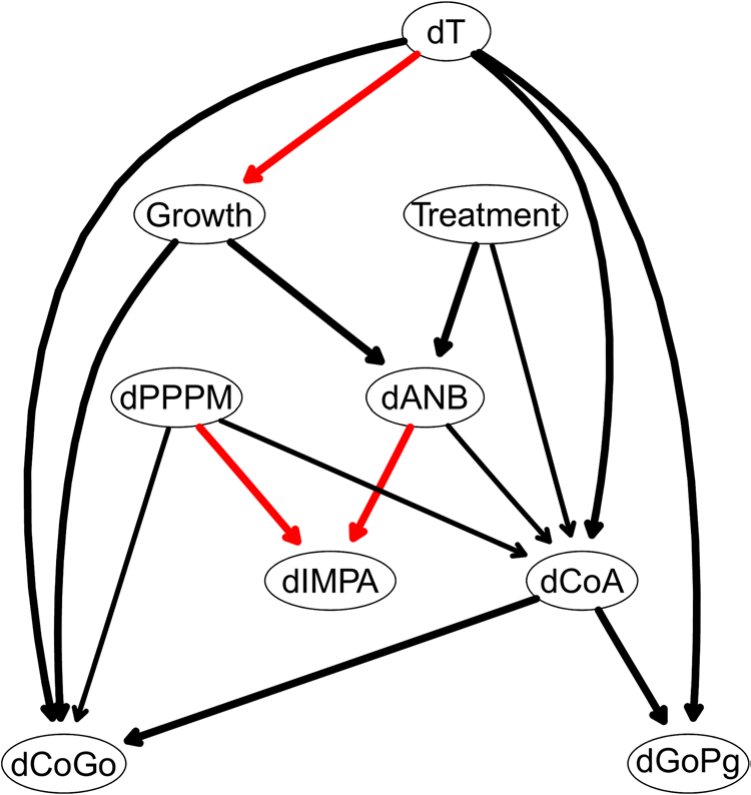
A simplified DAG derived from that in Fig. 2 after removing arcs with a strength smaller than 0.85.

The Bayesian network consensus model constructed by learning 200 networks from the data and keeping the arcs that appear at least 50% of the time (threshold estimated from the data) is shown in Fig. 2. All the directions of the arcs seem to be well established; this can probably be attributed to the use of a whitelist and a blacklist, as they force the directions of nearby arcs to cascade into place. Furthermore, a cursory examination of the arc strengths above the threshold confirms that 15 out of 18 arcs in the consensus network appear in fact with a frequency of at least 0.85. All arc directions are also clearly established (all frequencies are equal to 1). This allows to further simplify the consensus network as shown in Fig. 3 while losing little information in the process. While the skeletal growth process influences the evolution of sagittal maxillomandibular imbalance (${\rm{\Delta }}ANB$) and mandible ramps height (${\rm{\Delta }}CoGo$), the treatment effects mainly influence the maxillary length growth (${\rm{\Delta }}CoA$), and the progression of maxillomandibular imbalance (${\rm{\Delta }}ANB$).

To further validate the Bayesian network, we check whether it is consistent with prior information on Class III malocclusion that has not been used in the construction of the model. We formalise this prior information into four hypotheses, and we use conditional probability queries as described in the Methods (with 10^4^ random samples) to test them.

1. In Class III growing subjects an excessive growth of *CoGo* induces a reduction in *PPPM*, assuming no treatment is taking place. In the differential equations in the network, we have that as ${\rm{\Delta }}CoGo$ increases (which indicates an increasingly rapid growth) ${\rm{\Delta }}PPPM$ becomes increasingly negative (which indicates a reduction in the angle). This is shown in Fig. 4.

**Figure 4:**
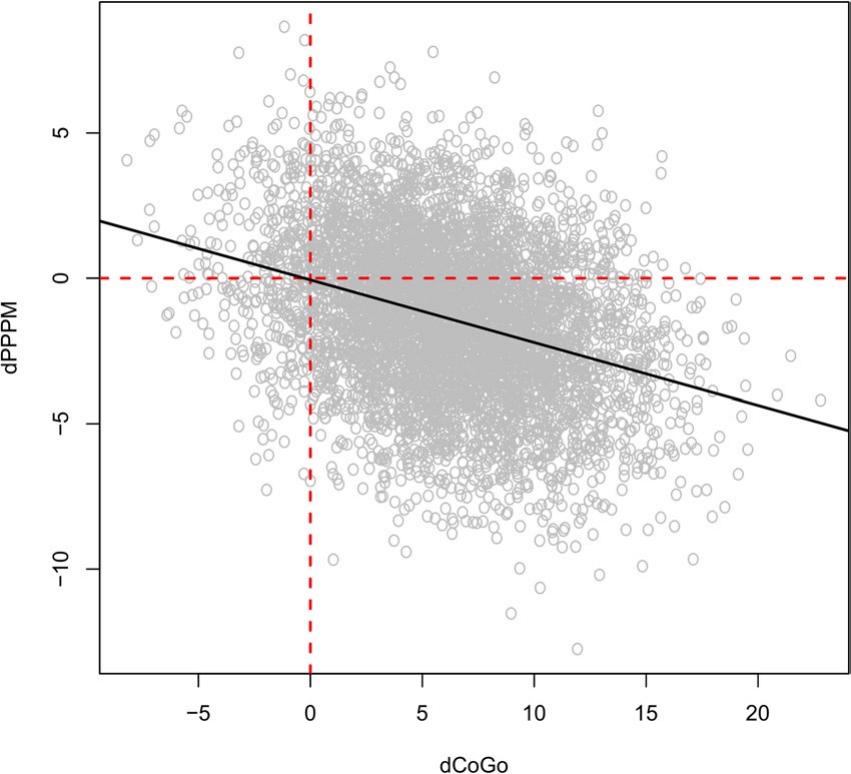
Values simulated from the Bayesian network for ${\rm{\Delta }}PPPM$ and ${\rm{\Delta }}CoGo$. The black line represents the regression line of ${\rm{\Delta }}PPPM$ against ${\rm{\Delta }}CoGo$; its negative slope confirms that as ${\rm{\Delta }}CoGo$ increases (which indicates an increasingly rapid growth) ${\rm{\Delta }}PPPM$ becomes increasingly negative (which indicates a reduction in the angle).
2. In Class III growing subjects, if *ANB* decreases *IMPA* decreases to compensate; ${\rm{\Delta }}ANB$ is proportional to ${\rm{\Delta }}IMPA$, as shown in Fig. 5, so a decrease in one suggests a decrease in the other.

**Figure 5:**
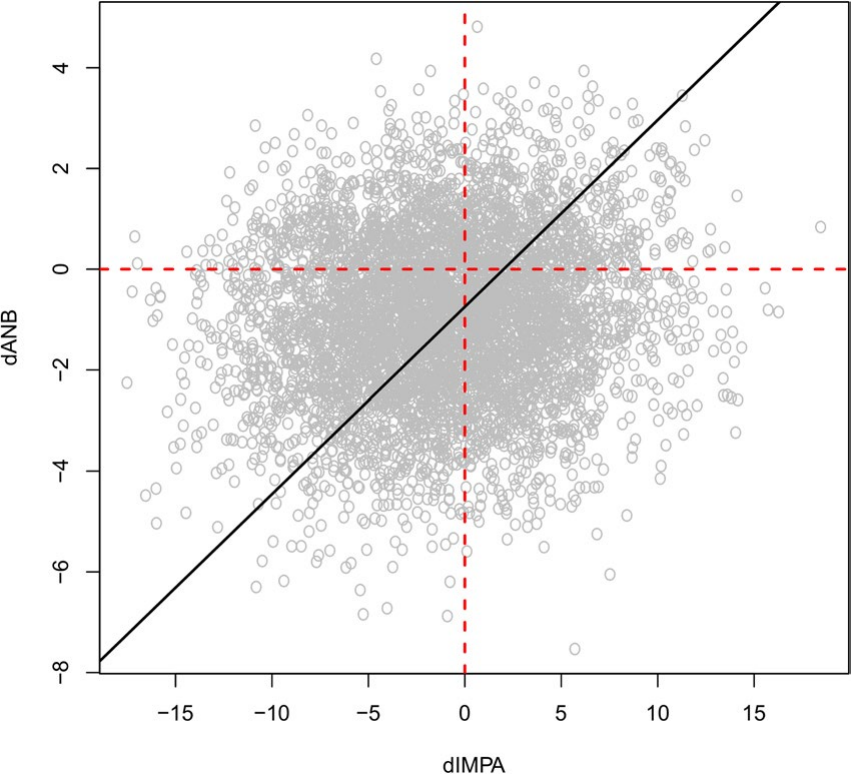
Values simulated from the Bayesian network for ${\rm{\Delta }}ANB$ and ${\rm{\Delta }}IMPA$. The black line represents the regression line of ${\rm{\Delta }}ANB$ against ${\rm{\Delta }}IMPA$; its positive slope suggests that ${\rm{\Delta }}ANB$ is proportional to ${\rm{\Delta }}IMPA$, so a decrease in one suggests a decrease in the other.
3. Since Class III orthodontic treatment is aimed at stopping the decrease of *ANB* (${\rm{\Delta }}ANB\approx 0$), we expect to observe different dynamics for *ANB* in treated and untreated patients. First, we note that the Bayesian network correctly assigns a higher probability of a favourable prognosis to treated (0.63) compared to untreated (0.51) patients. The unfavourable prognosis of treated patients was defined as the concurred presence of Class III permanent molar relationship and negative overjet^35^. If we simulate the treatment effect and fix ${\rm{\Delta }}ANB\approx 0$ (thus making it independent from its parents and removing the corresponding arcs), we have that the probability of a favourable prognosis is the same (0.58) for both treated and untreated patients and thus it does not depend on the treatment. This suggests that a favourable prognosis of a Class III malocclusion is determined mainly by preventing changes in *ANB*.
4. If we use *GoPg* as a proxy for point B, the treatment does not affect point B after controlling for point A: if we keep *GoPg* fixed (${\rm{\Delta }}GoPg\approx 0$) the angle between point A and point B (${\rm{\Delta }}ANB$) evolves differently for treated and untreated patients. On average, ${\rm{\Delta }}ANB$ increases for treated patients (+0.37 degrees; strongly negative values denote horizontal imbalance, so a positive rate of changes indicate a reduction in imbalance) and decreases for untreated patients (−1.13 degrees; the imbalance slowly worsens over time).

Finally, we also consider the predictive accuracy of the consensus Bayesian network. Using cross-validation as described in the Methods, we find that the prognosis is accurately predicted with probability 0.73. The predictive correlations for the craniofacial features are 0.86 for ${\rm{\Delta }}CoGo$, 0.91 for ${\rm{\Delta }}GoPg$, 0.92 for ${\rm{\Delta }}CoA$, 0.23 for ${\rm{\Delta }}IMPA$, 0.42 for ${\rm{\Delta }}PPPM$ and 0.65 for ${\rm{\Delta }}ANB$.

**Figure 6:**
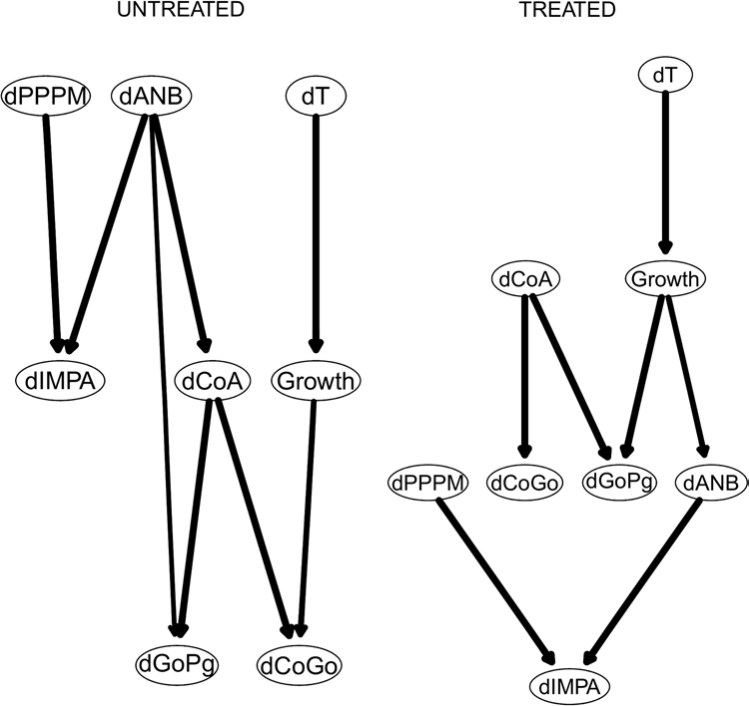
The DAGs underlying the consensus Bayesian networks for treated and untreated patients on the 8 variables measured for both, after adjusting them using the population reference values from Bathia and Leighton^41^.

We also learn two separate consensus Bayesian networks from treated and untreated patients, which are shown Fig. 6. There are significant differences between the influence networks pertaining to treated and untreated subjects. The treatment effects on the craniofacial subspaces are channelled through the maxillary node ${\rm{\Delta }}CoA$ to the mandibulary nodes ${\rm{\Delta }}CoGo$ (mandibular ramus) and ${\rm{\Delta }}GoPg$ (mandibular body). The adaptive ${\rm{\Delta }}IMPA$ node (which aims to maintain unchanged the sagittal relationship between the maxilla and the mandible) is influenced by both horizontal and vertical skeletal imbalances during the treatment process (i.e., by ${\rm{\Delta }}PPPM$ and ${\rm{\Delta }}ANB$). On the contrary, among Class III untreated subjects the progression of the horizontal skeletal imbalance (${\rm{\Delta }}ANB$) strictly influences the maxillary node ${\rm{\Delta }}CoA$, which in turn influences the progression of mandibulary nodes ${\rm{\Delta }}GoPg$ and ${\rm{\Delta }}CoGo$ during the growth process.

### Adjusted data

**Figure 7:**
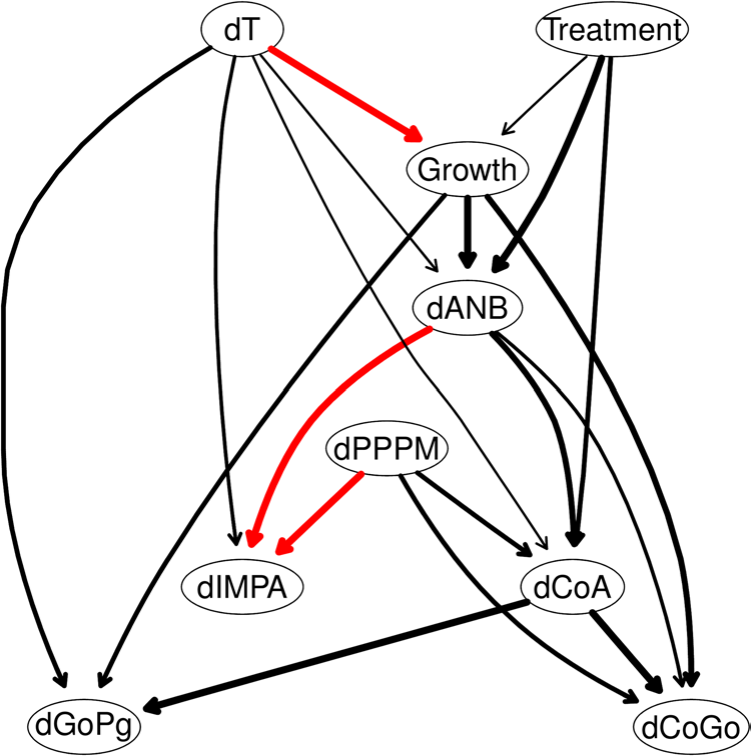
The DAG underlying the Bayesian network learned from the 9 variables measured on all 143 patients after adjusting them using the population reference values from Bathia and Leighton^41^.

**Figure 8:**
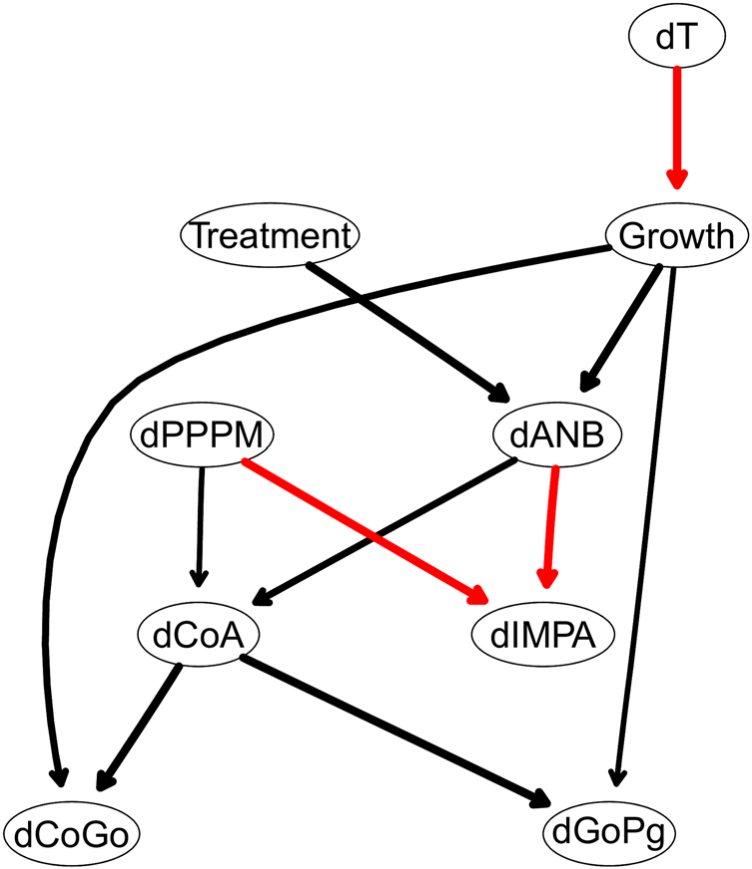
A simplified DAG derived from that in Fig. 7 after removing arcs with a strength smaller than 0.85.

With the aim of confirming the methodological approach we introduced in this paper, we considered another case study. This is obtained by adjusting the data by subtracting the corresponding reference values from an Atlas of normal cephalometric features^2^ for infancy and childhood. A consensus Bayesian networks built in the same way as that for the raw data is shown in Fig. 7. The threshold for the significance of the arcs is about the same as before (0.5) and the number of arcs is also similar. Again we can simplify the network by retaining only the arcs with an arc strength of at least 0.85. The most striking feature of this new network, shown in Fig. 8, is the absence of arcs between ${\rm{\Delta }}T$ and the orthodontic variables; the only arc from ${\rm{\Delta }}T$ points to *Growth* and is only included because of the whitelist. (Note that the same happens when we exclude the individuals with the most extreme ${\rm{\Delta }}T$, and the resulting networks in the two cases are very similar.) This seems to suggest that much of the dependence on ${\rm{\Delta }}T$ observed in the raw data is not a consequence of the evolution of the malocclusion but a result of ageing; and it is consistent with the fact that if we reduce the spread in the observed ages by removing the most extreme ${\rm{\Delta }}T$, most of the dependencies on ${\rm{\Delta }}T$ vanish. We conjecture that the nonlinear trend of the raw values of the orthodontic variables (that is, the fact that that their rate of change is a function of time) can be decomposed into two components: a general population average and a deviation from that average given the malocclusion. The former effectively changes with time (that is, the trend of the population mean over time is not constant) while the latter does not (that is, the rate of change of the deviation from the population mean depends only on other orthodontic values and on the treatment). In other words, the population average evolves with age for all orthodontic variables, which is expected as the patients are not yet fully grown adults. However, the deviations from the populations averages do not seem to evolve with age, or to be a function of the passage of time. This would imply that the effects applying a change to one of the orthodontic variables propagate to related orthodontic variables and cause them to respond them in the same way regardless of how quickly the change is applied. (e.g. a one-degree shift in *ANB* influences neighbouring variables such as *CoA* and *IMPA* in the same way regardless of how quickly that one-degree change happens; it can be one year, it can be two years, etc. but those neighbouring variables will have the same value at the end).

We consider the predictive accuracy of this new consensus Bayesian network, using cross-validation as we did for the network we learned from the raw data. We find that the probability of correctly predicting the prognosis is similar (0.74 vs 0.73). The predictive correlations for most craniofacial features, however, are smaller: 0.64 (−0.22) for ${\rm{\Delta }}CoGo$, 0.68 (−0.23) for ${\rm{\Delta }}GoPg$, 0.81 (−0.11) for ${\rm{\Delta }}CoA$, 0.28 (+0.05) for ${\rm{\Delta }}IMPA$, 0.39 (−0.03) for ${\rm{\Delta }}PPPM$ and 0.71 (+0.06) for ${\rm{\Delta }}ANB$. This is expected since we are now modelling deviations from the population average, which are intrinsically more difficult to analyse.

**Figure 9:**
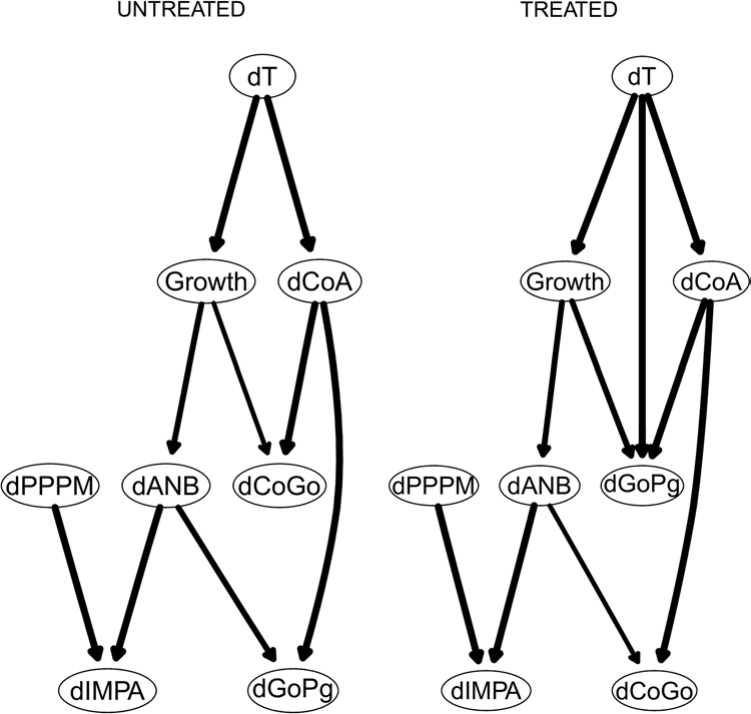
The DAGs underlying the consensus Bayesian networks for treated and untreated patients on the 8 variables measured for both.

We also learn two separate consensus Bayesian networks from treated and untreated patients, which are shown Fig. 9. Among both untreated and treated subjects the progression of skeletal imbalance is influenced by the evolution of the maxillary sagittal dimensions (${\rm{\Delta }}CoA$); however, only treated patients exhibit the strong dependency of the mandibulary corpus from ${\rm{\Delta }}CoA$.

## Discussion

Previous studies have proposed different cephalometric models to determine specific facial parameters related to abnormal growth patterns in Class III untreated and treated patients^2,4,5^. Multilevel, nonparametric, and predictive function algorithms have provided growth predictions and treatment outcomes based on a variety of facial characteristics^4,5^. Recently, network approaches to understanding morphological and functional relationships among orthodontic data have been proposed^16,17^. While these approaches improved the interpretation of quantitative, patient-specific information, networks were unable to elucidate the effects of influences (possibly, causal influences) between craniofacial variables^16^. Craniofacial features change and adapt as a system in response to both natural stimuli such as growth and external stimuli such as clinical treatments. This implies that efforts towards understanding diseases such as malocclusion must reflect this interplay in the choice of statistical models and in how clinically relevant hypotheses are tested. This has motivated the use of network approaches^16,17^ which represent features as nodes in an undirected graph and explicitly groups them into clusters based on their pair-wise correlation. These clusters can describe particular regions of the craniomaxillary and mandibular complexes and other broad features (symmetry, proportions, etc.) that are impacted by malocclusion and that must be targeted by treatment.

This modelling approach, however, has three important limitations. The first is that the use of pair-wise correlations makes it impossible to distinguish direct relationships between two features from indirect ones that are mediated by other features, thus making it difficult to get a clear picture of system as a whole and to identify the best target for the treatment^36^. Furthermore, the direction of the relationships is not taken into consideration by the model nor it is represented in the graph, making it impossible to infer cause-effect relationships even in the presence of data systematically collected from a clinical trial^13^. Finally, limiting the model to a representation of pair-wise dependencies falls short of characterising the full probability distribution of the features, and that in turn makes it impossible to use it to test the complex hypotheses required for model validation and treatment evaluation.

Bayesian networks suffer from none of these limitations^12^. Thanks to their modular model structure and to the availability of efficient software implementations^15^, they can be used to simultaneously explore a large number of features. The number of features does not impact the interpretability of the network: focusing on direct dependencies means that each feature is described by a local distribution that depends only those features (the “parents”) for which the corresponding nodes have an arc with an arrow pointing to that particular feature. Therefore, complex models are divided into a collection of simpler problems which are mathematically tractable and computationally simpler. Furthermore, the DAG can always be used as a high level abstraction for qualitative reasoning in the context of exploratory analysis and to investigate hypotheses on the whether various sets of features are related to each other^24^. Finally, a Bayesian network can also be interpreted as a causal network in the absence of confounding factors^13^ and used to examine or generate novel clinical hypotheses^37^. The inherently Bayesian nature of a Bayesian network facilitates such reasoning by incorporating both prior knowledge about the variables of interest and the uncertainty present in the data^38^; and by not defining model estimation and inference around a single response at the expense of the ability to reason about other variables (unlike, e.g., linear regression models).

However, information becomes knowledge only when it is placed in context: without it, the orthodontist has no way to differentiate signal from noise, so the research for better diagnosis and treatment might be swamped by false positives and false assumptions. Often in everyday practice the orthodontist’s efforts are aimed (or perhaps compelled) to the optimisation of therapy more than the optimisation of diagnosis. The result is that the therapy is effective, sometimes extraordinarily effective, but the price of a hasty diagnosis is paid in terms of relapse of the pretreatment craniofacial features. The aim of this work is therefore to obtain an integrated view of the craniofacial features, the treatment and the prognosis to allow systematic reasoning in the diagnostic process.

The use of Bayesian networks allows us to achieve this aim. We identify the focal morphological areas of the treatment for Class III malocclusion as the *CoA* segment (the maxillary length) and the *ANB* angle (the antero-posterior relation of the maxilla to the mandible); therefore, any apparent effect of the treatment on other cranial features can be disregarded as noise since it is actually mediated by these two features. Furthermore, by modelling the putative causal relationships we can study how the effect of an intervention on one feature propagates by identifying neighbouring features in the DAG and by studying how their distribution changes in response to various stimuli. We performed such an exercise to validate the consensus Bayesian network with respect to prior knowledge on malocclusion, with promising results.

To our knowledge, this is the first time a complex system such as craniofacial features has been modelled in this way with a formal statistical and causal Bayesian network. The usefulness of such a model is two-fold: it provides an intuitive qualitative description (in the form of a DAG) of the relationships that link the craniofacial features beyond mere physical proximity; and it also provides a quantitative description of their behaviour that can be used to validate the model and to test novel hypotheses by simulation. Collecting clinical data on either treated or untreated patients in the context of clinical trials is expensive, time consuming and subject to many practical, legal and deontological problems. In this context, Bayesian networks provide a way to perform a preliminary verification of the hypotheses that would be targeted by those trials to prioritise the trials and allocate resources efficiently^39^. For instance, in this paper we identified the focal point of the effect of the facemask therapy. To perform the same task experimentally without the help of the Bayesian network would require us to check many different locations; but with the indications provided by the Bayesian networks we can concentrate on *CoA* and *ANB* first and possibly avoid further experiments involving the remaining features. This sequential approach to experimental design and planning is becoming increasingly common in systems biology to reduce the cost of *in vitro* and *in vivo* research programmes^40^, and by the pharmaceutical industry to reduce the costs and risk of clinical trials^11^.

While Bayesian networks can deal with the uncertainty in the data, their main limitations lie in the impact of confounding variables and in the assumptions they make about the distribution of the features. With a larger number of measurements per patients, for instance, we expect that assuming linear relationships between the features would be a significant limitation, since we would have enough statistical power to detect nonlinear relationships.

The results of this study show that the Bayesian networks applied to a growing craniofacial complex are a useful tool to define a more detailed individualised prognosis for patients affected by the Class III malocclusion, and to mitigate an unpredictable ultimate outcome of this dysmorphosis.

## Electronic supplementary material

Supplementary information
