# Supplementary material for: Bayesian Networks Analysis of Malocclusion Data
Source: Sci Rep. 2017 Nov 10;7:15236. doi: 10.1038/s41598-017-15293-w (PMC5681542; doi:10.1038/s41598-017-15293-w)
Supplement: Supplementary file 1 — Supplementary information [file 41598_2017_15293_MOESM1_ESM.pdf]

Supplementary information: Bayesian Networks Analysis of Malocclusion Data

Marco Scutari[1], Pietro Auconi[2], Guido Caldarelli[3,4,5,*], and Lorenzo Franchi[6,7]

[1]Department of Statistics, University of Oxford, 24-29 St Giles' Oxford OX1 3LB, UK
[2]Private Practice of Orthodontics, Roma, Italy
[3]IMT School for Advanced Studies, Piazza San Francesco 19, 55100 Lucca, Italy
[4]Istituto dei Sistemi Complessi CNR, Unità Sapienza, Dip. Fisica , P.le A. Moro 2, 00185 Rome, Italy
[5]London Institute for Mathematical Sciences, 35a South St, Mayfair London W1K 2XF, UK
[6]Dipartimento di Ortodonzia, Università di Firenze, Firenze, Italy
[7]Center for Human Growth and Development, University of Michigan, Ann Arbor MI, USA
[*]Guido.Caldarelli@imtlucca.it

ABSTRACT

In this paper we use Bayesian networks to determine and visualise the interactions among various Class III malocclusion maxillofacial features during growth and treatment. We start from a sample of 143 patients characterised through a series of a maximum of 21 different craniofacial features. We estimate a network model from these data and we test its consistency by verifying some commonly accepted hypotheses on the evolution of these disharmonies by means of Bayesian statistics. We show that untreated subjects develop different Class III craniofacial growth patterns as compared to patients submitted to orthodontic treatment with rapid maxillary expansion and facemask therapy. Among treated patients the *CoA* segment (the maxillary length) and the *ANB* angle (the antero-posterior relation of the maxilla to the mandible) seem to be the skeletal subspaces that receive the main effect of the treatment.

1 Malocclusion Data Analysis

1.1 Data

The quantities under consideration are derived from the anatomy of the patient as shown in Fig. **??**.

For the data set of 143 patient, we have the following quantities with two measurements at ages T_1 and T_2 (measured in years):

- *ID*: anonymised ID code unique to each patient.
- *Treatment*: untreated ("NT"), treated with bad results ("TB"), treated with good results ("TG").
- *Growth*: a binary variable with values "Good" or "Bad", determined on the basis of CoGn-CoA.
- *ANB*: angle between Down's points A and B (degrees).
- *IMPA*: incisor-mandibular plane angle (degrees).
- *PPPM*: palatal plane - mandibular plane angle (degrees).
- *CoA*: total maxillary length from condilion to Down's point A (mm).
- *GoPg*: length of mandibular body from gonion to pogonion (mm).
- *CoGo*: length of mandibular ramus from condilion to pogonion (mm).

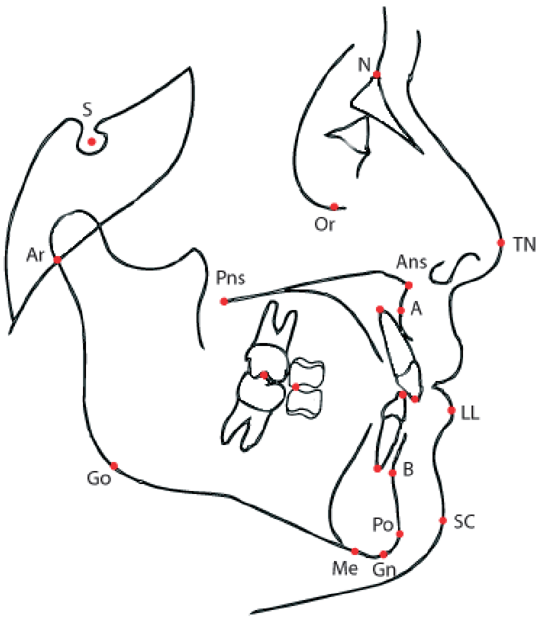

Figure 1. Cephalometric landmarks.
